# A Meta-Analysis and an Evaluation of Trends in Obesity Prevalence among Children and Adolescents in Turkey: 1990 through 2015

**DOI:** 10.4274/jcrpe.5043

**Published:** 2018-02-26

**Authors:** Züleyha Alper, İlker Ercan, Yeşim Uncu

**Affiliations:** 1 Uludağ University Faculity of Medicine, Department of Family Practice, Bursa, Turkey; 2 Uludağ University Faculity of Medicine, Department of Biostatistics, Bursa, Turkey

**Keywords:** Childhood, adolescence, obesity

## Abstract

**Objective:**

Obesity in childhood and adolescence is one of the most serious public health problems due to a remarkable increase in prevalence in recent years and its close relationship with non-communicable diseases, such as diabetes and hypertension, resulting in increased adult morbidity and mortality. This study aims to quantify the secular trend in different regions of Turkey from 1990 to 2015 by performing a meta-analysis of childhood and adolescent obesity prevalence studies conducted.

**Methods:**

Uludag University Library Database was searched for relevant articles published prior to March 2017. The heterogeneity of the studies in the meta-analysis was tested by the I2 statistic and Cochran’s Q test. The obesity trend analyses were examined by chi-square trend analysis with respect to five year periods. The statistical significance level was taken as α=0.05.

**Results:**

A total of 76 papers were initially identified addressing childhood and adolescent obesity in Turkey. Fifty-eight papers were selected for analysis. The prevalence of obesity increased from 0.6% to 7.3% with an 11.6-fold increase between the periods 1990-1995 to 2011-2015. The prevalence of obesity increased in both genders. However, boys were more likely to be obese than girls.

**Conclusion:**

Studies on obesity prevalence in the 5-19 age group in Turkey have gained importance, especially in the 2000s. While a remarkable number of prevalence studies, mostly regional, have been conducted between 2005-2011, a gradual decline was observed thereafter. Further national and population-based surveys on prevalence of obesity in children and adolescents are definitely needed in Turkey.

## What is already known on this topic?

Obesity in childhood and adolescence is one of the most serious public health problems due to a remarkable increase in prevalence in recent years and its close relationship with non-communicable diseases, such as diabetes and hypertension, resulting in increased adult morbidity and mortality.

## 

### What this study adds?

The results of this present study reveal that further national, population-based surveys on the prevalence of obesity in children and adolescents are definitely needed in Turkey.

## Introduction

Obesity in childhood and adolescence generally manifests itself in school years. Even when it does not continue into adulthood, it is correlated with increased adult morbidity and mortality by causing chronic disease states such as diabetes and hypertension(1). The prevalence of obesity has been increasing rapidly and because of these facts it is now one of the most serious public health problems for the 21^st^ century. The World Health Organization (WHO) has reported that the percentage of overweight children under five years increased from 5% in 2000 to 6% in 2010. This increase has been estimated to result in over 42 million (6.3%) of children being overweight in 2013. This problem currently affects many low and middle-income countries, and especially urban residential areas (2). The increasing trend of being overweight is a worldwide problem as the number of adults with a body mass index (BMI) of >25 kg/m^2^ increased from 29.8% to 36.9% in men and from 29.8% to 38.0% in women between the years 1980 and 2013 (3).

This is also true in Turkey where rapid changes in lifestyles, including dietary and physical activity habits have contributed to a remarkable increase in the prevalence of obesity which is now accepted as a serious threat to public health. Turkish studies have reported different results for the prevalence of obesity in children and adolescents, depending upon geographic and cultural differences. According to the latest national representative data, the obesity prevalence in the 6-18 age group is 8.2% overall. The difference between genders and for area of residence are 7.3% for girls, 9.1% for boys and 9.7% in urban, 4.5% in rural settings respectively (4). Adoption of a Western lifestyle amongst children and living in an urban setting in a developing country are considered probable risk factors (4,5).

Prevalence Studies of Diabetes, Hypertension, Obesity and Endocrine Diseases in Turkey data have confirmed the scale of the public health problem associated with childhood obesity. Notably, the results show that as the education level of women rises, the obesity risk decreases independent of other factors, a finding which emphasizes the importance of the education of girls in improving community health status (6). 

The primary aim of this study is to identify the secular trend in the prevalence of childhood and adolescent obesity by performing a meta-analysis of studies conducted in different regions of the country between 1990 and 2015. We also aim to review the prevalence and changing trends of obesity among Turkish school children aged 5-19 years.

## Methods

### Search Strategy

The Uludağ University Library Database was searched to identify relevant papers in Turkish and in English published prior to March 2017 (7). The following key words were used: [‘incidence’ OR ‘frequency’ OR ‘prevalence’ OR ‘epidemiology’] AND [‘obesity’ OR ‘body mass index’ OR ‘BMI’ OR ‘weight gain’] AND [‘Turkey’ OR ‘Turkish’] AND [‘childhood’ OR ‘children’ OR ‘adolescence’ OR ‘adolescents’ OR ‘youth’ OR ‘teen’ OR ‘teenager’] for obesity in Turkish children and adolescents.

Studies were selected according to the following criteria:

i) A sample that included school children (5-19 years of age),

ii) Cross-sectional design,

iii) Original studies on prevalence of obesity,

iv) Studies conducted within the borders of Turkey,

v) Studies that defined obesity categories according to BMI calculated by dividing body weight (kg) by the square of height (m^2^) and those that used the age and sex specific BMI percentile tables by Neyzi et al (8), or those of the centers for disease control and prevention (9) or WHO (10).

Studies which lacked sufficient data, or were repetitive studies based on the same database were considered as not meeting the inclusion criteria and were excluded.

**Figure 1:**
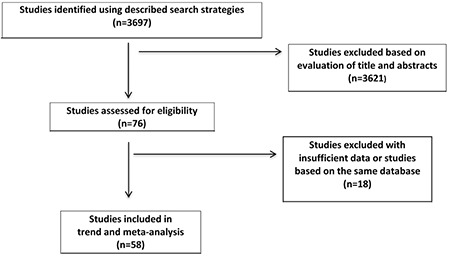
Flow chart for selection of studies for inclusion in this trend and meta-analysis for obesity

Figure 1 summarizes the flow chart for selection of studies for inclusion in this meta-analysis for obesity.

### Data Extraction

On the basis of pre-defined inclusion criteria, titles and abstracts were examined for inclusion by two independent reviewers (ZA and YU) and disagreements were resolved by consensus or, if necessary, by referral to a third reviewer (IE). The full text forms were evaluated for the articles with titles and/or abstracts with insufficient information. Publication year; study time, period and place; study design; representativeness of target population; sample selection; sample size; data source; data collection; description of obesity; sex; age; study objectives; criteria for obesity; and figures that allowed calculation of obesity prevalence were extracted from the studies. We assessed the quality of all included studies on the basis of the following: study design, representativeness of target population, sample selection, sample size, response rate, data source and study objectives, data collection, description of obesity, sex, and age. Studies were rated (++) if all or most of checklist criteria were fulfilled; (+) if some criteria were fulfilled; and (-) if few or no criteria were fulfilled. All data extractions were ratified by one researcher (IE). Missing raw data were requested from authors by email or by phone calls.

### Statistical Analysis

A meta-analysis was made for determining the summary statistics oriented towards the prevalence of obesity. The heterogeneity of the studies in the meta-analysis was tested by the I^2^ statistic and Cochran’s Q test. In the heterogeneity test, a was taken as 0.10. For the estimation of the summary statistics, the fixed effect model in case of homogeneity and the random effect model in the contrary case were used. The publication bias was assessed by inspection of Funnel plots. Statistics concerning the meta-analysis results are given in tables and by Forest plots.

The obesity trend analyses were examined by chi-square trend analysis with respect to 5-year periods, as there were insufficient studies conducted on a yearly basis. Thus, five blocks of 5-year periods were defined as 1990-1995, 1996-2000, 2001-2005, 2006-2010 and 2011-2015 and compared. In the trend analysis, instead of publication year, the year in which the field study was performed was used. For the studies in which the exact research periods were not stated, the necessary information was obtained by communicating with the author via e-mail and by phone. Any studies in which precise information was not available concerning the year the research was made were excluded from the research. By taking the 1990-1995 period as a baseline, the statistical significance levels for the next 5-year periods and the odds ratio values were calculated. The statistical significance level was taken as a=0.05.

### Ethics

Information reported in this retrospective study was collected by references to published works. Ethical responsibility is related to the authors of the studies made. 

## Results

The analysis included studies which were conducted in different cities and regions of Turkey on school children aged between 5-19 years. While evaluating each of the studies one by one in the meta-analysis, in the trend analysis we evaluated the total of the studies made in different regions in the same 5-year periods, instead of representing only one location, with the aim of evaluating the trends in obesity in the 5-year periods. When all of the studies done between the years 1990-1995 were used as a baseline, we observed that there was an increase in the prevalence of obesity in the following 5-year periods. 

**Figure 2:**
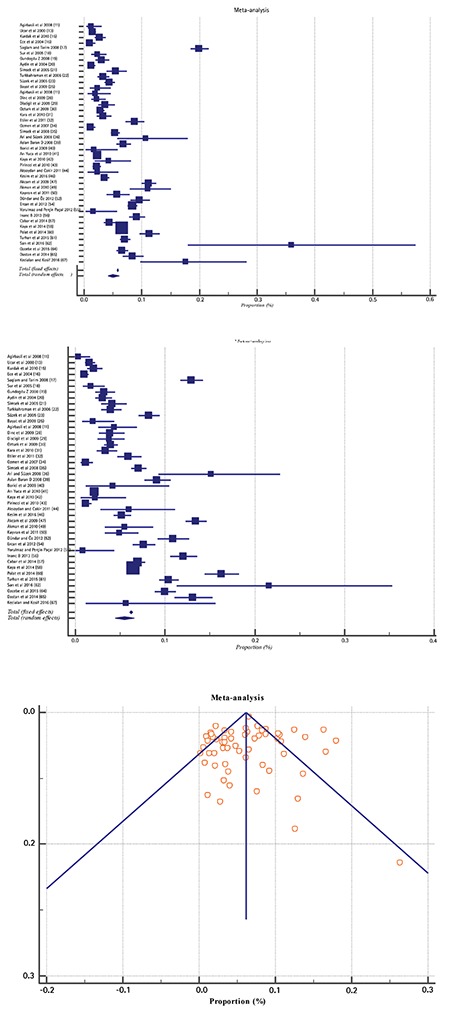
Pooled analysis for obesity in children and adolescents in Turkey, overall, girls and boys respectively

After screening 76 papers, we included 58 papers in the analysis. Figure 2 shows the Forest and Funnel plots of 58 studies of obesity prevalence with a total number of subjects of 230 252 Turkish school children aged 5-19 years to evaluate overall obesity prevalence. For assessing gender specific obesity prevalence 43 papers with a total of 100 086 girls and 108 491 boys aged 5-19 years were assessed. The prevalence of obesity was found as 5.7% [95% confidence interval (CI), 4.8-6.6] totally, 5.0% (95% CI, 4.1-6.1) in girls and 5.5% (95% CI, 4.4-6.6) in boys (Table 1). 

Time trend analyses based on data collection years showed that obesity increased 11.6-fold (5.8-fold for girls, 24.5-fold for boys) from 1990-1995 to 2011-2015. The prevalence of obesity increased in both genders, but boys were more likely to be obese than girls (Table 2). 

**Figure 3:**
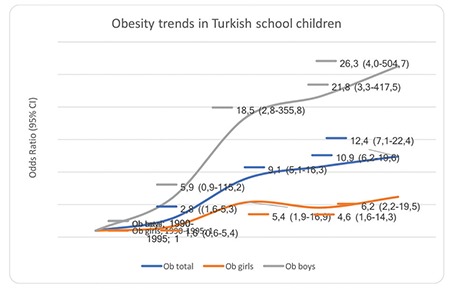
Trends in obesity prevalence [OR (95% CI)] in Turkish children and adolescents aged 5-19 years from 1990-1995 through 2011-2015. Data obtained from all 58 publications with and without gender discrimination

**Figure 4:**
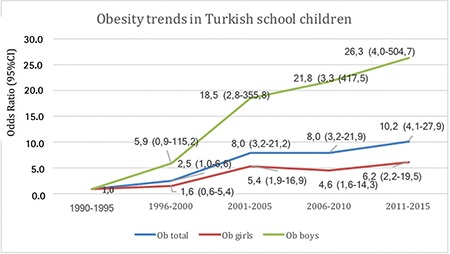
Trends in obesity prevalence [OR (95% CI)] in Turkish children and adolescents aged 5-19 years from 1990-1995 through 2011-2015. Data obtained from 43 publications in which the data are given separately as overall. girls and boys

While the prevalence of obesity increased from 0.7% to 7.1% between 1990-1995 and 2011-2015 (for girls: 1.2% to 6.8%; for boys: 0.3% to 7.4%) according to the data obtained from 43 publications in which the data are given separately as overall, girls and boys, we observed that the overall prevalence of obesity increased from 0.6% to 7.3% by analysing the trend with 58 publications. (Table 2,3) (Figure 3,4).

## Discussion

This meta-analysis indicates that the prevalence of obesity has increased significantly among both girls and boys in Turkey since 1990 and that this increase is much more marked in boys. 

In the present study, we consulted 58 different studies conducted on prevalence of obesity between 1990 and 2015 in school children aged 5-19. All studies confirmed an increase in obesity, though the magnitude of this increase varied. 

### Study Limitations

The meta-analysis reported here combines data across studies conducted in different cities and groups in Turkey in order to estimate trends in obesity in school children aged 5-19 with more precision than is possible in a single study. The main limitations of this meta-analysis. as with any overview. are the differences between the age groups of the study population. insufficient age-specific data and regional and cultural differences. Among these studies. there were publications whose aim was not to determine obesity prevalence and publications which did not discriminate between obesity prevalence according to gender. despite the fact that they were well designed. So. the quality of the data cannot go beyond the quality of the individual studies included and the results can only be representative of the studies that have been included and are unable to provide a representation of all studies published.

## Conclusion

However, the results of this present study reveal that further national. regular population-based surveys representing Turkey on the prevalence of obesity in children and adolescents are definitely needed. We. as authors. wish to increase awareness of this global public health concern in order to develop comprehensive public health policies and strategies to improve the prevention and management of obesity and related diseases. We also wish to provide baseline data for monitoring the effectiveness of national programs for control of obesity in the future. which we suggest should be a high priority public health initiative for Turkey. It should not be forgotten that obesity. and obesity-related non-communicable diseases. will negatively affect immediate health. quality of life and educational attainment in childhood and adolescence and will likely have a permanent negative effect on the future life of the child. 

## Figures and Tables

**Table 1:**
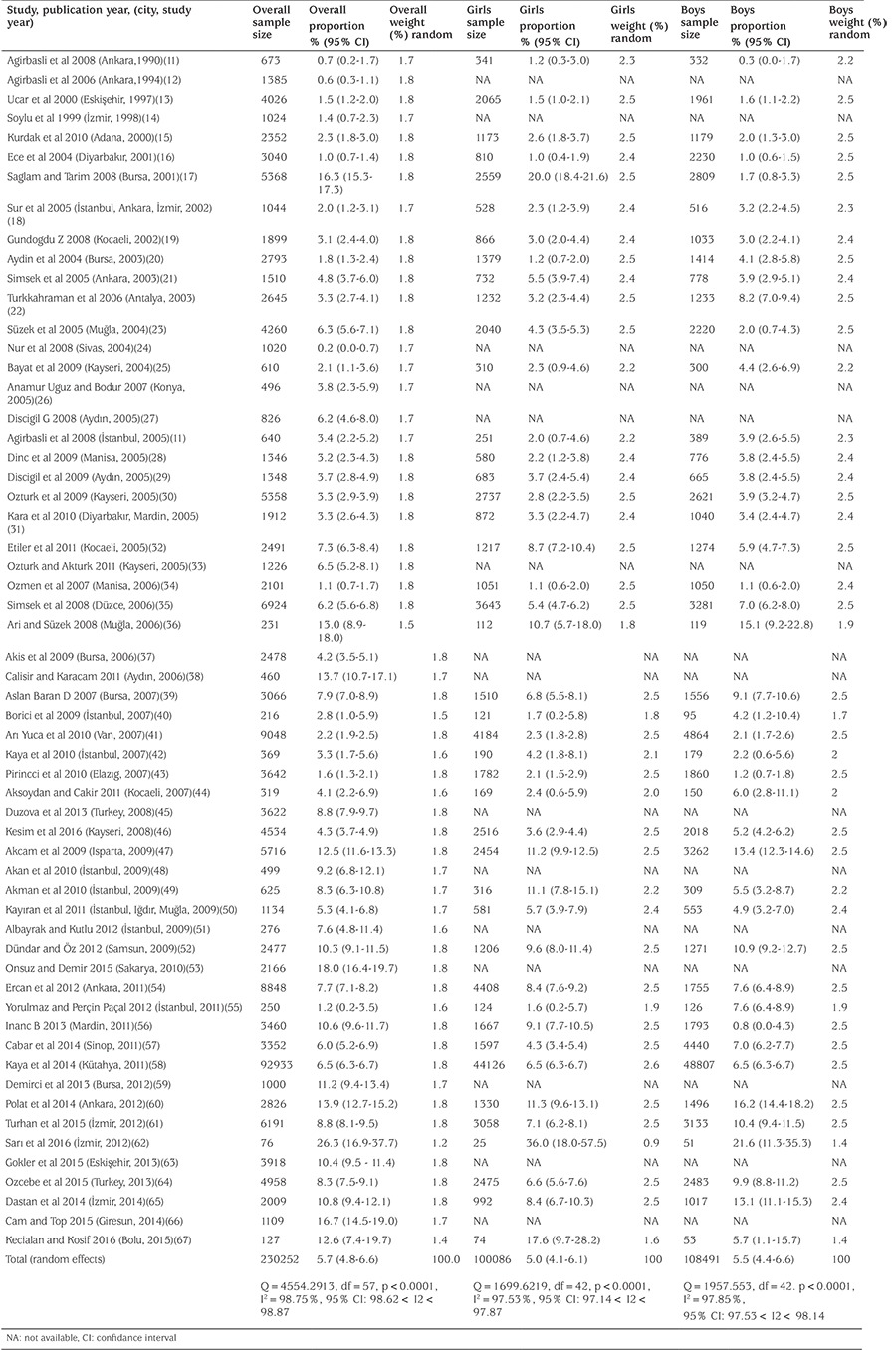
Meta-analysis for obesity in children and adolescents in Turkey

**Table 2:**
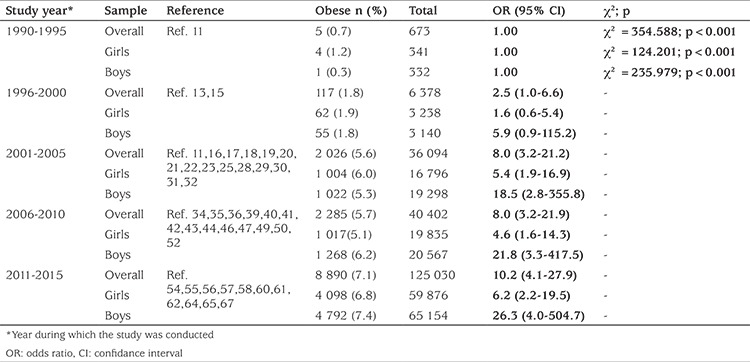
Trend analysis for obesity in children and adolescents in Turkey

**Table 3:**
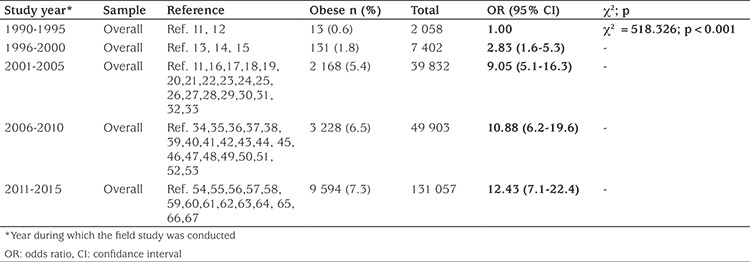
Trend analysis for obesity in children and adolescents in Turkey
